# Supplementary material for: Comprehensive Annotation and Functional Exploration of MicroRNAs in Lettuce
Source: Front Plant Sci. 2021 Dec 24;12:781836. doi: 10.3389/fpls.2021.781836 (PMC8739914; doi:10.3389/fpls.2021.781836)
Supplement: Supplementary Data 1 — Results of categories 0–2 from PARE-Seq analysis (including three subfiles:1_1, 1_2, 1_3). [file Data_Sheet_10.ZIP › GSM2230755.plot/Lsa-miR2111_Lsat_1_v5_gn_9_118540.1_116_TPlot.pdf]

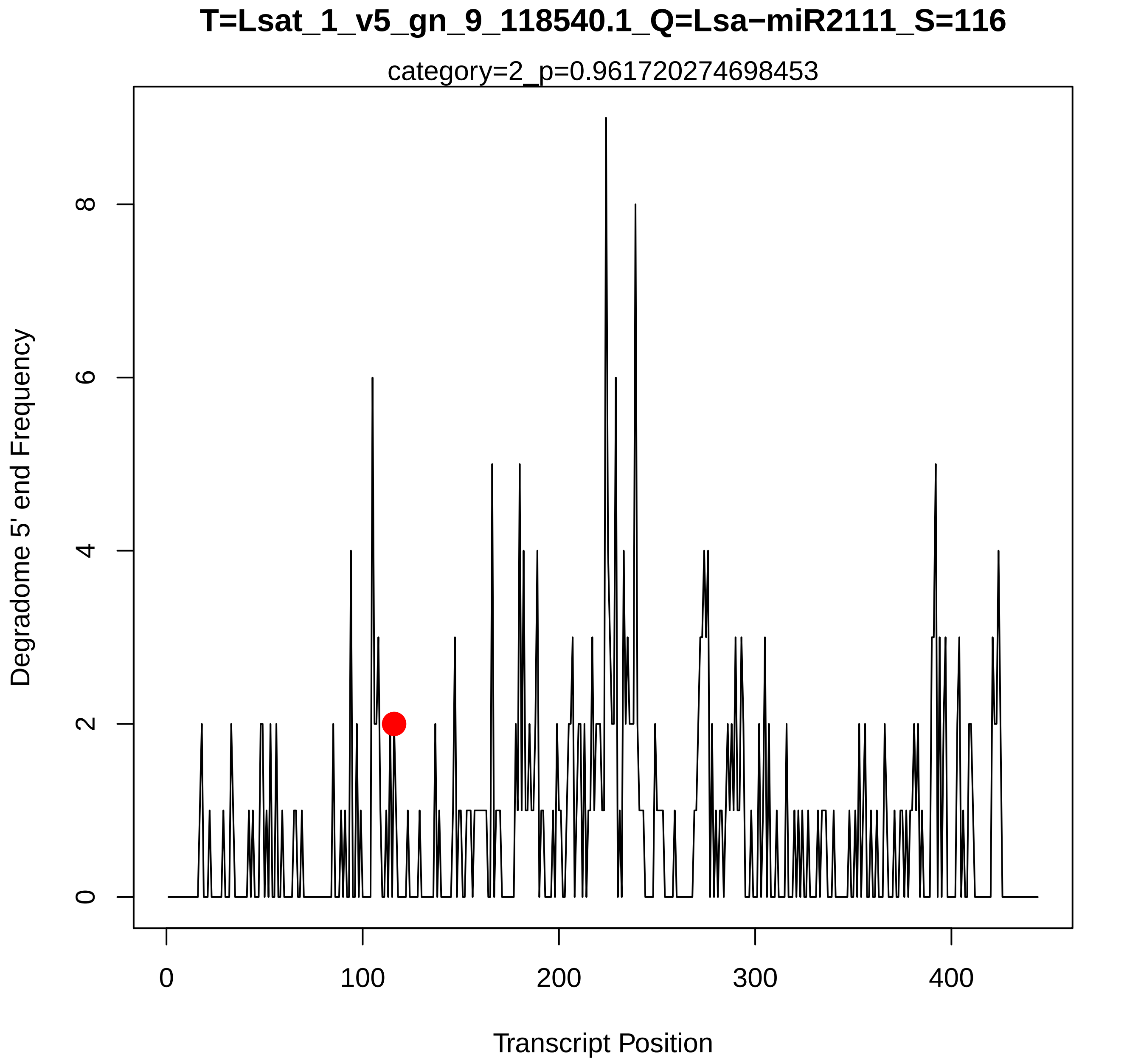

T=Lsat_1_v5_gn_9_118540.1_Q=Lsa−miR2111_S=116
category=2_p=0.961720274698453
Degradome 5' end Frequency
0
2
4
6
8
0
100
200
300
400
Transcript Position
